# Crystal structure and Hirshfeld surface analysis of a Schiff base: (*Z*)-6-[(5-chloro-2-meth­oxy­anilino)methyl­idene]-2-hy­droxy­cyclo­hexa-2,4-dien-1-one

**DOI:** 10.1107/S2056989019002123

**Published:** 2019-02-12

**Authors:** Sibel Demir Kanmazalp, Onur Erman Doĝan, Volkan Taşdemir, Necmi Dege, Erbil Aĝar, Igor O. Fritsky

**Affiliations:** a Gaziantep University, Technical Sciences, 27310, Gaziantep, Turkey; b Ondokuz Mayıs University, Faculty of Arts and Sciences, Department of Chemistry, 55139, Kurupelit, Samsun, Turkey; c Science Research and Applied Center, Van Yuzuncu Yil University, 65080, Van, Turkey; d Ondokuz Mayıs University, Faculty of Arts and Sciences, Department of Physics, 55139, Kurupelit, Samsun, Turkey; e Department of Chemistry, Taras Shevchenko National University of Kyiv, Volodymyrska 64/13, 01601 Kyiv, Ukraine

**Keywords:** crystal structure, Schiff bases, Hirshfeld surface, hydrogen bonds, stacking inter­actions

## Abstract

The title compound, C_14_H_12_ClNO_3_, is a Schiff base that exists in the keto–enamine tautomeric form and adopts a *Z* configuration. In the crystal, the dihedral angle between the planes of the benzene rings is 5.34 (15)°. The roughly planar geometry of the mol­ecule is stabilized by a strong intra­molecular N—H⋯O hydrogen bond. In the crystal, pairs of centrosymmetrically related mol­ecules are linked by O—H⋯O hydrogen bonds, forming *R*_2_^2^(10) rings. Besides this, the mol­ecules form stacks along the [001] direction with C—H⋯π and C—H⋯Cl contacts between the stacks. The inter­molecular inter­actions in the crystal were analysed using Hirshfeld surfaces. The most significant contribution to the crystal packing is from H⋯H contacts (30.8%).

## Abstract 2

The title compound, C_14_H_12_ClNO_3_, adopts the keto–enamine tautomeric form. In the crystal, mol­ecules form stacks along the [001] direction. The crystal packing is further stabilized by O—H⋯O and C—H⋯O hydrogen bonds and C—H⋯Cl and C—H⋯π contacts.

## Chemical context   

Schiff bases are widely used as ligands in coordination chemistry (Calligaris & Randaccio, 1987 ▸) and they are also of inter­est in various fields because of their diverse biological activity (Lozier *et al.*, 1975 ▸; Costamagna *et al.*, 1992 ▸). Some Schiff bases derived from salicyl­aldehyde have attracted the inter­est of chemists and physicists because they show thermo­chromism and photochromism in the solid state (Cohen *et al.*, 1964 ▸; Hadjoudis *et al.*, 1987 ▸). The origin of their photo- and thermochromism is related to the reversible intra­molecular proton transfer associated with a change in the electronic structure (Hadjoudis *et al.*, 1987 ▸). The *o*-hy­droxy Schiff bases obtained by the condensation of *o*-hy­droxy­aldehydes with aniline have been extensively examined in this context. Such compounds can exist in two tautomeric forms, *viz.* keto–enamine (N—H⋯O) and phenol–imine (N⋯H—O) (Stewart & Lingafelter, 1959 ▸; Petek *et al.*, 2010 ▸). We report herein the synthesis and the crystal and mol­ecular structures of the title compound, as well as an analysis of its Hirshfeld surfaces.
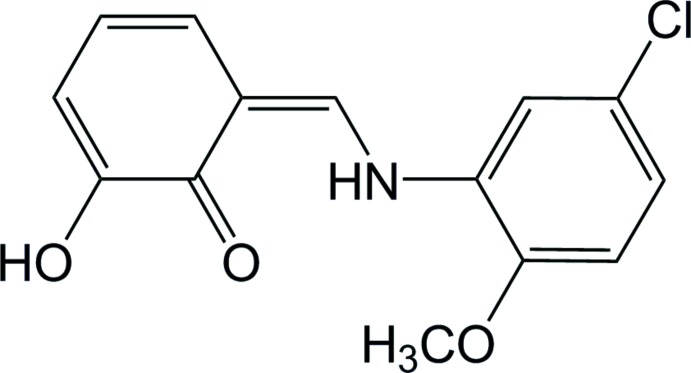


## Structural commentary   

**Figure 1:**
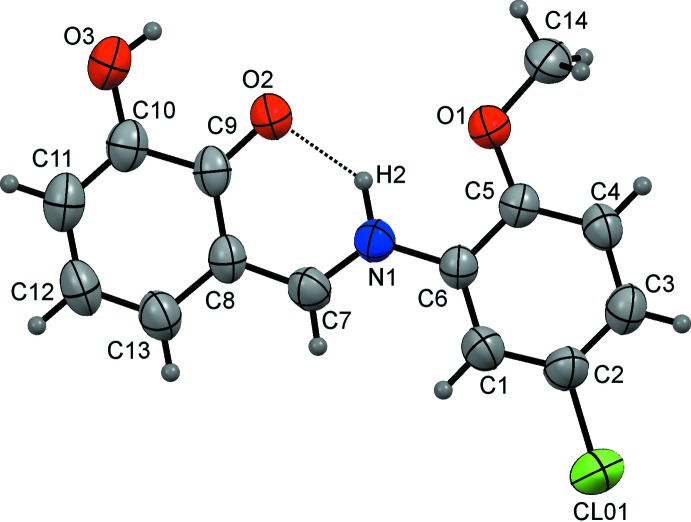
The mol­ecular structure of the title compound with the displacement ellipsoids drawn at the 50% probability level. The intra­molecular N—H⋯O hydrogen bond is shown as a dashed line.

As shown in Fig. 1 ▸., the asymmetric unit of the title compound contains only one mol­ecule, which adopts the keto–enamine tautomeric form: the H atom is located at N1, and the lengths of the N1—C7 and C8—C9 bonds indicate their single-bond character, whereas the O2—C9 and C7—C8 bonds are double (Table 1 ▸). Overall, the bond lengths in the title structure compare well with those of other keto–enamine tautomers known from the literature (see the *Database survey* section). The whole mol­ecule is almost planar, with a dihedral angle of 5.34 (15)° between the benzene ring planes. The meth­oxy C14 atom deviates from the plane of the C1–C6 benzene ring by 0.038 (4) Å. The torsion angles C1—C6—N1—C7 and N1—C7—C8—C9 are 5.8 (5) and −0.6 (5)°, respectively. The planar mol­ecular conformation is stabilized by the intra­molecular N1—H2⋯O2 hydrogen bond (Table 2 ▸).

## Supra­molecular features   

**Figure 2:**
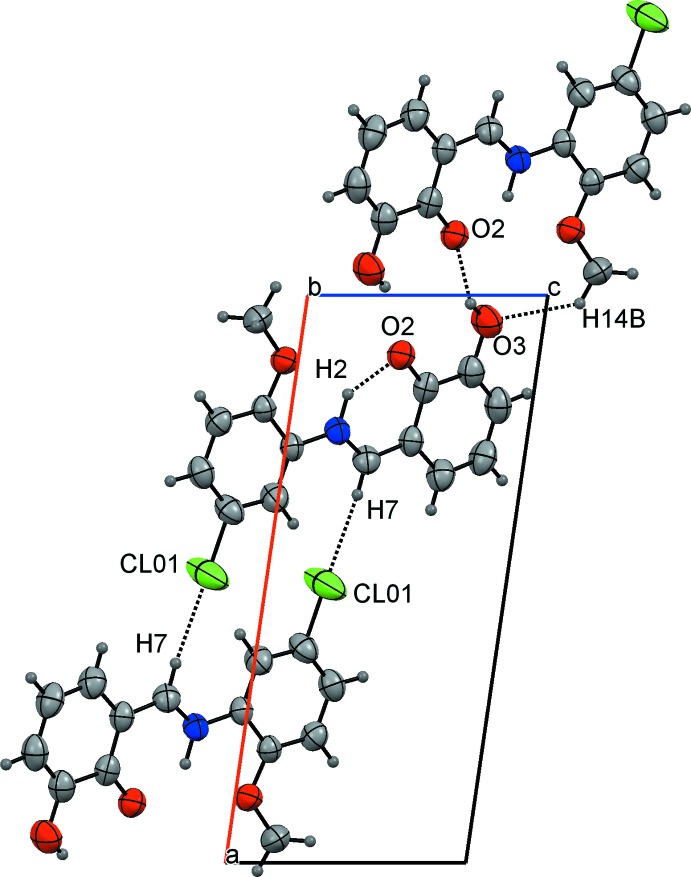
A view of the crystal packing of the title compound. Dashed lines denote the intra- and inter­molecular hydrogen bonds.

**Figure 3:**
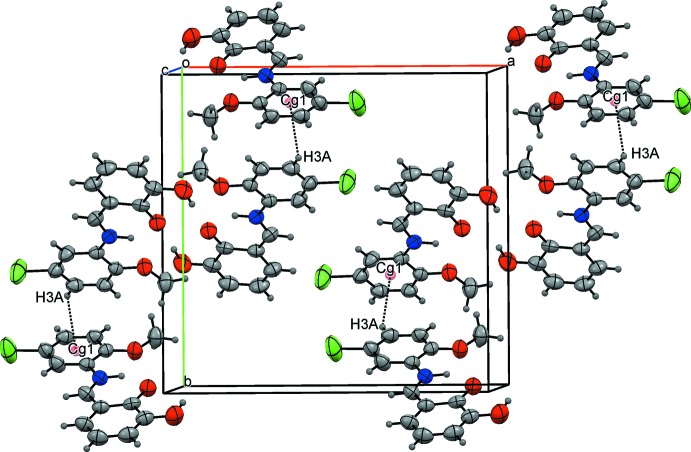
The packing diagram showing the stacking, C—H⋯π and C—H⋯Cl inter­actions.

In the crystal, the mol­ecules are connected *via* O—H⋯O hydrogen bonds into centrosymmetric pairs with an 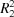
(10) graph-set motif (Table 2 ▸, Fig. 2 ▸). Mol­ecules related by a [001] translation form stacks with an inter­planar distance of 3.420 (3) Å and a shortest inter­centroid separation of 3.6797 (17) Å. The mol­ecular packing is further stabilized by C—H⋯O, C—H⋯Cl and C—H⋯π inter­actions between the mol­ecules of the neighbouring stacks (Fig. 3 ▸). Details of all these contacts are given in Table 2 ▸.

## Database survey   

A search of the Cambridge Structural database (CSD, version 5.40, update November 2018; Groom *et al.*, 2016 ▸) for the 3-[(*E*)-(phenyl­imino)­meth­yl]-benzene-1,2-diol fragment revealed eight hits where this fragment adopts the keto–enamine tautomeric form and 21 hits where it exists as the phenol–imine tautomer. Distinctive bond lengths (N1—C7, C7=C8, C8—C9, C9=O2) in the title structure are the same within standard uncertainties as the corresponding bond lengths in the structures of 2-hy­droxy-6-[(2-meth­oxy­phen­yl)amino­methyl­ene]cyclo­hexa-2,4-dienone (FOCCOQ; Şahin *et al.*, 2005 ▸) and 6-[(4-chloro­phenyl­amino)­methyl­ene]-2,3-di­hydroxy­cyclo­hexa-2,4-dien-1-one (CIRTED; Karabıyık *et al.*, 2008 ▸). In the structures of typical phenol–imine tautomers, *viz.*, 3-[(3-bromo­phen­yl)imino­meth­yl]benzene-1,2-diol (CUCZUW; Keleşoğlu *et al.*, 2009*b* ▸), 3-[(2-bromo­phen­yl)imino­meth­yl]benzene-1,2-diol (XEYSOK; Temel *et al.*, 2007 ▸) and 3-[(4-butyl­phen­yl)imino­meth­yl]benzene-1,2-diol (XOZJUS; Keleşoğlu *et al.*, 2009*a* ▸), the C9—O2 and C7—C8 bond lengths are distinctly longer, being in the ranges 1.324–1.355 Å and 1.427–1.447 Å, respectively. It is likely that the inter­molecular O—H⋯O hydrogen bond, where the keto O atom acts as an hydrogen-bond acceptor, is an important prerequisite for the tautomeric shift toward the keto–enamine form. In fact, in all eight structures of the keto–enamine tautomers, hydrogen bonds of this type are observed. However, in 16 of 21 structures of phenol–imine tautomers, such hydrogen bonds are also present. This means that there is another unknown reason for the formation of keto–enamine tautomers.

## Hirshfeld surface analysis   

**Figure 4:**
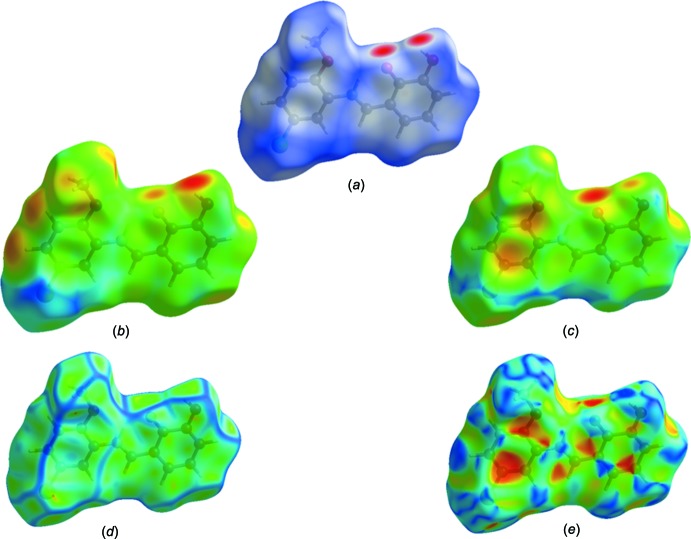
The Hirshfeld surface of the title compound mapped with (*a*) *d_norm_*, (*b*) *d_i_*, (*c*) *d_e_*, (*d*) curvedness and (*e*) shape-index.

**Figure 5:**
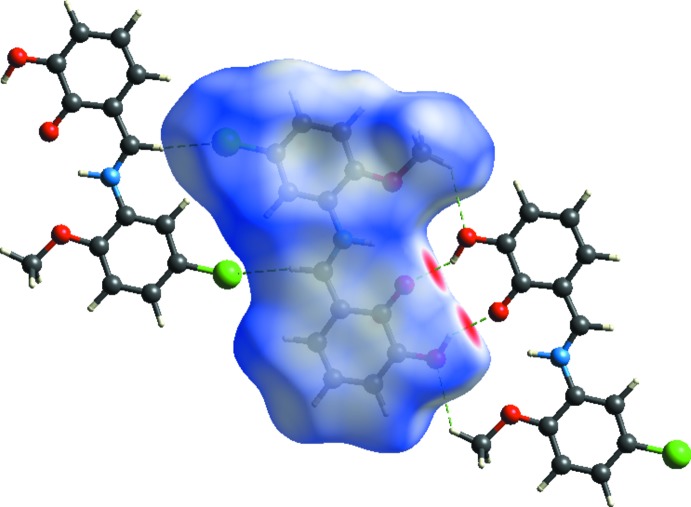
The *d*_norm_-mapped Hirshfeld surface showing the inter­molecular inter­actions in the title compound.

**Figure 6:**
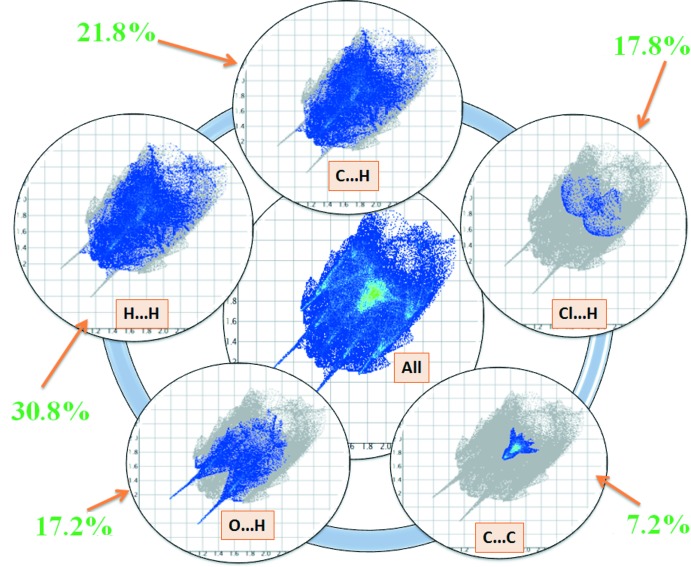
Two-dimensional fingerprint plots with *d_norm_* views of all, the H⋯H, O⋯H/H⋯O, C⋯H/H⋯C and N⋯H/H⋯N contacts in the title compound.

**Figure 7:**
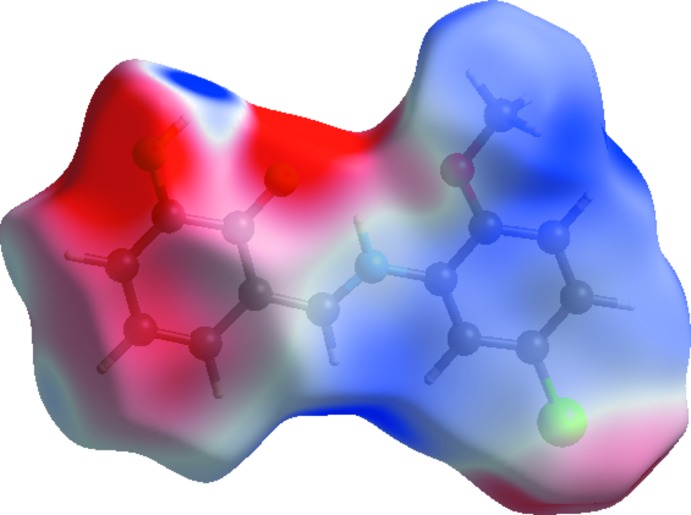
The view of the Hirshfeld surface of the title compound plotted over the electrostatic potential energy.

The Hirshfeld surface analysis, together with the two-dimensional fingerprint plots, is a powerful tool for the visualization and inter­pretation of inter­molecular contacts in mol­ecular crystals, since it provides a concise description of all inter­molecular inter­actions present in a crystal structure (Spackman & Jayatilaka, 2009 ▸; McKinnon *et al.*, 2007 ▸). All surfaces and 2D fingerprint plots were generated using *CrystalExplorer3.1* (Wolff *et al.*, 2012 ▸). The mappings of *d_i_*, *d_e_*, *d_norm_*, shape-index and curvedness for the title structure are shown in Fig. 4 ▸. The Hirshfeld surface of a mol­ecule in the crystal is presented in Fig. 5 ▸, with the prominent hydrogen-bonding inter­actions shown as intense red spots. The two-dimensional fingerprint plots provide information about the percentage contributions of the various inter­atomic contacts. As can be seen from these plots (Fig. 6 ▸), the most important are the H⋯H inter­actions, which contribute 30.8% to the total Hirshfeld surface. Other contributions are from O⋯C/C⋯O (1.2%), O⋯H/H⋯O (17.2%), C⋯C (7.2%), O⋯O/O⋯O (1.0%), Cl⋯H/H⋯Cl (17.8%) and C⋯H/H⋯C (21.8%). Analogous features were observed recently for some compounds of the same class (Kansız *et al.*, 2018 ▸; Özek Yıldırım *et al.*, 2018 ▸). The donor and acceptor centers of the hydrogen bonding are represented as blue (positive) and red (negative) regions on the Hirshfeld surface mapped over the electrostatic potential (Fig. 7 ▸). The electrostatic potential of the Cl01 atom is less negative as compared to those of atoms O2 and O3 of the hy­droxy groups, as indicated by the lighter red color.

## Synthesis and crystallization   

The title compound was prepared by mixing solutions of 2,3-di­hydroxy­benzaldehyde (34.5 mg, 0.25 mmol) and 5-chloro-2-meth­oxy­aniline (39.4 mg, 0.25 mmol), both in 15 mL of ethanol, with subsequent stirring for 5 h under reflux. Single crystals were obtained by slow evaporation of an ethanol solution (yield 65%; m.p. 442–444 K).

## Refinement   

Crystal data, data collection and structure refinement details are summarized in Table 3 ▸. The C-bound H atoms were geometrically positioned with C—H distances of 0.93–0.96 Å and refined as riding, with *U*_iso_(H) = 1.2*U*_eq_(C) or *U*_iso_(H) = 1.5*U*_eq_(C) for methyl H atoms. The O- and N-bound H atoms were located in a difference map and freely refined.

## Supplementary Material

Crystal structure: contains datablock(s) I. DOI: 10.1107/S2056989019002123/yk2119sup1.cif

Structure factors: contains datablock(s) I. DOI: 10.1107/S2056989019002123/yk2119Isup2.hkl

Supporting information file. DOI: 10.1107/S2056989019002123/yk2119Isup3.cml

CCDC reference: 1886956

Additional supporting information:  crystallographic information; 3D view; checkCIF report

## Figures and Tables

**Table 1:** Selected bond lengths (Å)

O2—C9	1.292 (4)	C8—C13	1.410 (5)
O3—C10	1.358 (4)	C9—C10	1.426 (4)
N1—C6	1.413 (4)	C10—C11	1.359 (5)
N1—C7	1.302 (4)	C11—C12	1.402 (5)
C7—C8	1.408 (4)	C12—C13	1.349 (4)
C8—C9	1.429 (4)		

**Table 2:** Hydrogen-bond geometry (Å, °) *Cg*1 is the centroid of the C1–C6 ring.

*D*—H⋯*A*	*D*—H	H⋯*A*	*D*⋯*A*	*D*—H⋯*A*
C7—H7⋯Cl01^i^	0.93	2.88	3.737 (3)	154
C14—H14*B*⋯O3^ii^	0.96	2.59	3.295 (4)	131
O3—H3⋯O2^ii^	0.87 (4)	2.00 (4)	2.780 (4)	148 (4)
N1—H2⋯O2	0.97 (4)	1.82 (4)	2.598 (3)	136 (4)
C3—H3*A*⋯*Cg*1^iii^	0.93	2.73	3.463 (3)	136

Symmetry codes: (i) 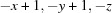; (ii) 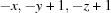; (iii) 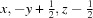.

**Table 3:** Experimental details

Crystal data	
Chemical formula	C_14_H_12_ClNO_3_
*M*_r_	277.70
Crystal system, space group	Monoclinic, *P*2_1_/*c*
Temperature (K)	296
*a*, *b*, *c* (Å)	14.7251 (9), 14.4444 (9), 6.1698 (4)
β (°)	98.241 (5)
*V* (Å^3^)	1298.74 (14)
*Z*	4
Radiation type	Mo *K*α
μ (mm^−1^)	0.30
Crystal size (mm)	0.23 × 0.16 × 0.09

Data collection	
Diffractometer	Stoe IPDS 2
Absorption correction	Integration (*X-RED32*; Stoe & Cie, 2002 ▸)
*T*_min_, *T*_max_	0.948, 0.979
No. of measured, independent and observed [*I* > 2σ(*I*)] reflections	13658, 2491, 1120
*R*_int_	0.115
(sin θ/λ)_max_ (Å^−1^)	0.617

Refinement	
*R*[*F* ^2^ > 2σ(*F* ^2^)], *wR*(*F* ^2^), *S*	0.057, 0.100, 0.90
No. of reflections	2491
No. of parameters	181
H-atom treatment	H atoms treated by a mixture of independent and constrained refinement
Δρ_max_, Δρ_min_ (e Å^−3^)	0.16, −0.24

Computer programs: *X-AREA* and *X-RED* (Stoe & Cie, 2002 ▸), *SHELXT* (Sheldrick, 2015*a* ▸), *SHELXL2014* (Sheldrick, 2015*b* ▸), *ORTEP-3 for Windows* and *WinGX* (Farrugia, 2012 ▸) and *PLATON* (Spek, 2009 ▸).

## supplementary crystallographic information

### Crystal data

**Table:**

C_14_H_12_ClNO_3_	*F*(000) = 576
*M**_r_* = 277.70	*D*_x_ = 1.420 Mg m^−^^3^
Monoclinic, *P*2_1_/*c*	Mo *K*α radiation, λ = 0.71073 Å
*a* = 14.7251 (9) Å	Cell parameters from 8086 reflections
*b* = 14.4444 (9) Å	θ = 1.4–27.1°
*c* = 6.1698 (4) Å	µ = 0.30 mm^−^^1^
β = 98.241 (5)°	*T* = 296 K
*V* = 1298.74 (14) Å^3^	Irregular specimen, red
*Z* = 4	0.23 × 0.16 × 0.09 mm

### Data collection

**Table:**

Stoe IPDS 2 diffractometer	2491 independent reflections
Radiation source: sealed X-ray tube, 12 x 0.4 mm long-fine focus	1120 reflections with *I* > 2σ(*I*)
Detector resolution: 6.67 pixels mm^-1^	*R*_int_ = 0.115
rotation method scans	θ_max_ = 26.0°, θ_min_ = 2.0°
Absorption correction: integration (X-RED32; Stoe & Cie, 2002)	*h* = −18→18
*T*_min_ = 0.948, *T*_max_ = 0.979	*k* = −17→17
13658 measured reflections	*l* = −7→7

### Refinement

**Table:**

Refinement on *F*^2^	Primary atom site location: structure-invariant direct methods
Least-squares matrix: full	Secondary atom site location: difference Fourier map
*R*[*F*^2^ > 2σ(*F*^2^)] = 0.057	Hydrogen site location: mixed
*wR*(*F*^2^) = 0.100	H atoms treated by a mixture of independent and constrained refinement
*S* = 0.90	*w* = 1/[σ^2^(*F*_o_^2^) + (0.0293*P*)^2^] where *P* = (*F*_o_^2^ + 2*F*_c_^2^)/3
2491 reflections	(Δ/σ)_max_ < 0.001
181 parameters	Δρ_max_ = 0.16 e Å^−^^3^
0 restraints	Δρ_min_ = −0.24 e Å^−^^3^

### Special details

**Table:**

Geometry. All esds (except the esd in the dihedral angle between two l.s. planes) are estimated using the full covariance matrix. The cell esds are taken into account individually in the estimation of esds in distances, angles and torsion angles; correlations between esds in cell parameters are only used when they are defined by crystal symmetry. An approximate (isotropic) treatment of cell esds is used for estimating esds involving l.s. planes.

### Fractional atomic coordinates and isotropic or equivalent isotropic displacement parameters (Å^2^)

**Table:**

	*x*	*y*	*z*	*U*_iso_*/*U*_eq_	
Cl01	0.49115 (8)	0.36774 (9)	−0.2476 (2)	0.1172 (6)	
O1	0.11503 (15)	0.37573 (16)	−0.0556 (4)	0.0611 (6)	
O2	0.10563 (15)	0.50349 (16)	0.4247 (4)	0.0601 (7)	
O3	0.0461 (2)	0.5981 (2)	0.7618 (4)	0.0782 (9)	
N1	0.2392 (2)	0.46609 (18)	0.2056 (5)	0.0497 (8)	
C1	0.3568 (2)	0.4173 (2)	−0.0160 (6)	0.0608 (10)	
H1A	0.4026	0.4475	0.0774	0.073*	
C2	0.3782 (2)	0.3706 (2)	−0.1958 (6)	0.0606 (10)	
C3	0.3124 (2)	0.3262 (2)	−0.3362 (6)	0.0583 (10)	
H3A	0.3277	0.2961	−0.4592	0.070*	
C4	0.2231 (2)	0.3264 (2)	−0.2937 (6)	0.0540 (9)	
H4	0.1780	0.2953	−0.3869	0.065*	
C5	0.2002 (2)	0.3725 (2)	−0.1142 (5)	0.0459 (8)	
C6	0.2675 (2)	0.4193 (2)	0.0256 (5)	0.0454 (8)	
C7	0.2902 (2)	0.5189 (2)	0.3445 (6)	0.0546 (9)	
H7	0.3515	0.5266	0.3277	0.066*	
C8	0.2572 (2)	0.5648 (2)	0.5188 (5)	0.0495 (9)	
C9	0.1632 (2)	0.5551 (2)	0.5481 (5)	0.0483 (9)	
C10	0.1348 (3)	0.6045 (2)	0.7269 (6)	0.0577 (9)	
C11	0.1941 (3)	0.6591 (2)	0.8593 (6)	0.0669 (11)	
H11	0.1733	0.6919	0.9723	0.080*	
C12	0.2862 (3)	0.6667 (2)	0.8274 (6)	0.0680 (11)	
H12	0.3262	0.7037	0.9205	0.082*	
C13	0.3171 (3)	0.6206 (2)	0.6623 (6)	0.0619 (10)	
H13	0.3784	0.6257	0.6430	0.074*	
C14	0.0424 (2)	0.3301 (3)	−0.1933 (6)	0.0698 (11)	
H00F	0.0378	0.3546	−0.3392	0.105*	
H14B	−0.0144	0.3402	−0.1375	0.105*	
H14C	0.0548	0.2649	−0.1959	0.105*	
H3	0.016 (3)	0.555 (3)	0.685 (8)	0.104 (18)*	
H2	0.175 (3)	0.461 (3)	0.226 (8)	0.130 (17)*	

### Atomic displacement parameters (Å^2^)

**Table:**

	*U*^11^	*U*^22^	*U*^33^	*U*^12^	*U*^13^	*U*^23^
Cl01	0.0754 (8)	0.1458 (11)	0.1426 (12)	−0.0326 (7)	0.0573 (8)	−0.0628 (9)
O1	0.0504 (14)	0.0746 (16)	0.0578 (16)	−0.0021 (13)	0.0062 (12)	−0.0180 (13)
O2	0.0581 (15)	0.0667 (16)	0.0544 (17)	−0.0025 (13)	0.0051 (13)	−0.0148 (12)
O3	0.077 (2)	0.087 (2)	0.074 (2)	0.0025 (16)	0.0234 (17)	−0.0277 (16)
N1	0.0535 (19)	0.0501 (19)	0.047 (2)	−0.0040 (14)	0.0114 (17)	−0.0032 (14)
C1	0.063 (3)	0.060 (2)	0.061 (3)	−0.0134 (17)	0.014 (2)	−0.0135 (18)
C2	0.061 (2)	0.057 (2)	0.068 (3)	−0.011 (2)	0.025 (2)	−0.010 (2)
C3	0.073 (3)	0.050 (2)	0.056 (3)	0.001 (2)	0.019 (2)	−0.0059 (19)
C4	0.060 (3)	0.048 (2)	0.053 (2)	−0.0013 (17)	0.005 (2)	−0.0097 (17)
C5	0.051 (2)	0.043 (2)	0.044 (2)	0.0025 (17)	0.0085 (18)	0.0000 (17)
C6	0.055 (2)	0.041 (2)	0.041 (2)	−0.0018 (16)	0.0093 (19)	−0.0023 (15)
C7	0.059 (2)	0.052 (2)	0.053 (2)	−0.0125 (18)	0.010 (2)	−0.0031 (19)
C8	0.063 (2)	0.042 (2)	0.043 (2)	−0.0008 (16)	0.0073 (19)	−0.0044 (16)
C9	0.067 (2)	0.0347 (19)	0.042 (2)	0.0064 (17)	0.004 (2)	−0.0009 (16)
C10	0.072 (3)	0.050 (2)	0.052 (2)	0.007 (2)	0.011 (2)	−0.0005 (19)
C11	0.093 (3)	0.056 (3)	0.053 (3)	0.007 (2)	0.015 (2)	−0.0073 (19)
C12	0.088 (3)	0.058 (2)	0.055 (3)	−0.011 (2)	0.000 (2)	−0.015 (2)
C13	0.074 (2)	0.054 (2)	0.057 (3)	−0.010 (2)	0.008 (2)	−0.0087 (19)
C14	0.053 (2)	0.095 (3)	0.060 (3)	−0.002 (2)	0.002 (2)	−0.010 (2)

### Geometric parameters (Å, º)

**Table:**

Cl01—C2	1.739 (3)	C4—H4	0.9300
O1—C5	1.354 (3)	C5—C6	1.393 (4)
O1—C14	1.429 (4)	C7—C8	1.408 (4)
O2—C9	1.292 (4)	C7—H7	0.9300
O3—C10	1.358 (4)	C8—C9	1.429 (4)
O3—H3	0.87 (4)	C8—C13	1.410 (5)
N1—C6	1.413 (4)	C9—C10	1.426 (4)
N1—C7	1.302 (4)	C10—C11	1.359 (5)
N1—H2	0.97 (4)	C11—C12	1.402 (5)
C1—C2	1.372 (4)	C11—H11	0.9300
C1—C6	1.376 (4)	C12—C13	1.349 (4)
C1—H1A	0.9300	C12—H12	0.9300
C2—C3	1.363 (5)	C13—H13	0.9300
C3—C4	1.379 (4)	C14—H00F	0.9600
C3—H3A	0.9300	C14—H14B	0.9600
C4—C5	1.375 (4)	C14—H14C	0.9600
			
C5—O1—C14	117.9 (3)	C8—C7—H7	118.3
C10—O3—H3	113 (3)	C7—C8—C13	119.7 (3)
C7—N1—C6	126.1 (3)	C7—C8—C9	119.8 (3)
C7—N1—H2	116 (3)	C13—C8—C9	120.4 (3)
C6—N1—H2	118 (3)	O2—C9—C10	120.3 (3)
C2—C1—C6	119.8 (3)	O2—C9—C8	123.2 (3)
C2—C1—H1A	120.1	C10—C9—C8	116.5 (3)
C6—C1—H1A	120.1	O3—C10—C11	119.6 (3)
C3—C2—C1	121.4 (3)	O3—C10—C9	118.9 (4)
C3—C2—Cl01	118.9 (3)	C11—C10—C9	121.4 (3)
C1—C2—Cl01	119.7 (3)	C10—C11—C12	120.8 (3)
C2—C3—C4	119.2 (3)	C10—C11—H11	119.6
C2—C3—H3A	120.4	C12—C11—H11	119.6
C4—C3—H3A	120.4	C13—C12—C11	120.3 (4)
C5—C4—C3	120.4 (3)	C13—C12—H12	119.8
C5—C4—H4	119.8	C11—C12—H12	119.8
C3—C4—H4	119.8	C12—C13—C8	120.5 (3)
O1—C5—C4	125.1 (3)	C12—C13—H13	119.7
O1—C5—C6	115.0 (3)	C8—C13—H13	119.7
C4—C5—C6	119.9 (3)	O1—C14—H00F	109.5
C1—C6—C5	119.3 (3)	O1—C14—H14B	109.5
C1—C6—N1	123.7 (3)	H00F—C14—H14B	109.5
C5—C6—N1	117.0 (3)	O1—C14—H14C	109.5
N1—C7—C8	123.3 (3)	H00F—C14—H14C	109.5
N1—C7—H7	118.3	H14B—C14—H14C	109.5
			
C6—C1—C2—C3	0.3 (6)	C6—N1—C7—C8	179.0 (3)
C6—C1—C2—Cl01	−179.7 (3)	N1—C7—C8—C13	179.9 (3)
C1—C2—C3—C4	−1.5 (6)	N1—C7—C8—C9	−0.6 (5)
Cl01—C2—C3—C4	178.6 (3)	C7—C8—C9—O2	2.0 (5)
C2—C3—C4—C5	1.3 (5)	C13—C8—C9—O2	−178.5 (3)
C14—O1—C5—C4	−1.8 (5)	C7—C8—C9—C10	−179.3 (3)
C14—O1—C5—C6	179.2 (3)	C13—C8—C9—C10	0.1 (5)
C3—C4—C5—O1	−178.8 (3)	O2—C9—C10—O3	−1.5 (5)
C3—C4—C5—C6	0.1 (5)	C8—C9—C10—O3	179.8 (3)
C2—C1—C6—C5	1.0 (5)	O2—C9—C10—C11	179.8 (3)
C2—C1—C6—N1	−179.5 (3)	C8—C9—C10—C11	1.2 (5)
O1—C5—C6—C1	177.8 (3)	O3—C10—C11—C12	179.7 (3)
C4—C5—C6—C1	−1.2 (5)	C9—C10—C11—C12	−1.7 (6)
O1—C5—C6—N1	−1.7 (4)	C10—C11—C12—C13	0.9 (6)
C4—C5—C6—N1	179.3 (3)	C11—C12—C13—C8	0.4 (6)
C7—N1—C6—C1	5.8 (5)	C7—C8—C13—C12	178.6 (3)
C7—N1—C6—C5	−174.7 (3)	C9—C8—C13—C12	−0.9 (5)

### Hydrogen-bond geometry (Å, º)

Cg1 is the centroid of the C1–C6 ring.

**Table:**

*D*—H···*A*	*D*—H	H···*A*	*D*···*A*	*D*—H···*A*
C7—H7···Cl01^i^	0.93	2.88	3.737 (3)	154
C14—H14*B*···O3^ii^	0.96	2.59	3.295 (4)	131
O3—H3···O2^ii^	0.87 (4)	2.00 (4)	2.780 (4)	148 (4)
N1—H2···O2	0.97 (4)	1.82 (4)	2.598 (3)	136 (4)
C3—H3*A*···*Cg*1^iii^	0.93	2.73	3.463 (3)	136

Symmetry codes: (i) −*x*+1, −*y*+1, −*z*; (ii) −*x*, −*y*+1, −*z*+1; (iii) *x*, −*y*+1/2, *z*−1/2.
